# Transcriptome Analysis of Two Different Developmental Stages of *Paeonia lactiflora* Seeds

**DOI:** 10.1155/2017/8027626

**Published:** 2017-08-07

**Authors:** Yonglei Ma, Jinqiu Cui, Xiujun Lu, Lijie Zhang, Zhijing Chen, Riwen Fei, Xiaomei Sun

**Affiliations:** Forestry College, Shenyang Agricultural University, Shenyang, Liaoning 110866, China

## Abstract

*Paeonia lactiflora* is a herbaceous flower in the family *Paeoniaceae* with both hypocotyl and epicotyl dormant seeds. We used high-throughput transcriptome sequencing on two different developmental stages of *P. lactiflora* seeds to identify seed dormancy and germination-related genes. We performed de novo assembly and annotated a total of 123,577 unigenes, which encoded 24,688 putative proteins with 47 GO categories. A total of 10,714 unigenes were annotated in the KEGG database, and 258 pathways were involved in the annotations. A total of 1795 genes were differentially expressed in the functional enrichment analysis. The key genes for seed germination and dormancy, such as *GAI1* and *ARF*, were confirmed by quantitative reverse transcription-polymerase chain reaction analysis. This is the first report of sequencing the *P. lactiflora* seed transcriptome. Our results provide fundamental frame work and technical support for further selective breeding and cultivation of *Paeonia*. Our transcriptomic data also serves as the basis for future genetics and genomics research on *Paeonia* and its closely related species.

## 1. Introduction

*Paeonia lactiflora* is a herbaceous perennial flower plant in the family *Paeoniaceae*, which is native to Central and Eastern Asia and widely grown in China. *Paeonia lactiflora* is one of horticulturally important flower species. It has been primarily grown for in use in the horticultural industry as home garden plants and is also cultivated as commercially cut flower. Furthermore, *P. lactiflora*, as a temperament plant species, is highly cold resistant and can normally grow and bloom under temperature −46.5°C. Therefore, *P. lactiflora* plants have not only become the main source of peonies for the cut flower business but they are also valuable cold-resistant genetic resources for breeding and cultivation [1].

Plant seeds usually experience dormancy where they are unable to germinate in a specified period of time. Seed dormancy is a very important mechanism to inhibit germination during unsuitable ecological conditions, for example, low temperature [2]. It has been known that dormancy is caused by two categories of factors: exogenous and endogenous. Exogenous factors include physical barriers of impermeable seed coat, which prevent the seeds from taking up water or gases. As results, the seed is unable to germinate until the physical impermeable layer is broken [3]. Endogenous dormancy is caused by embryonic conditions. For example, physiological immature embryos, lack of growth hormone, or presence of inhibiting chemicals all can retard embryo growth and prevent seed germination [4, 5]. In *P. lactiflora*, for example, abscisic acid (ABA) has been identified to be one of the major endogenous physiological factors to inhibit seed germination and root growth [6].

*Paeonia lactiflora* seeds display both hypocotyl and epicotyl dormancy and time from sowing to fully germination takes six to seven months under natural conditions. Hypocotyls of *P. lactiflora* start to elongate and stimulate the root growth when temperature goes down in later fall after sowing. After experiencing long winter, the dormancy of epicotyl is broken and starts to grow during spring [7]. Furthermore, incomplete removal of dormancy during seed reproduction leads to a decrease in germination rate. Therefore, such long process of dormancy and low germination rate greatly slow the *Paeonia lactiflora* breeding and cultivation. Due to these dual and sequential dormancy scenarios in *P. lactiflora*, understanding the hypocotyl dormancy is fundamental to tackle the epicotyl dormancy and further to unravel the dormancy of *P. lactiflora* [8]. Previous studies have been mainly focused on morphological and physiological perspective of seed dormancy in *P. lactiflora.* However, molecular mechanism of seed dormancy in *P. lactiflora* remains unexplored.

Previous studies have demonstrated that many genes regulate seed dormancy and germination and especially genes involving in ABA and gibberellic acid (GA) pathway [9–11]. Using whole genomic and transcriptomics analyses in *Arabidopsis*, numerous genes with various functions have been identified and shown differential expression between dormant seeds and dormancy-releasing seeds [12, 13]. To better understand the regulatory mechanisms and identify the genes underlying seed dormancy in *P. lactiflora*, which display dual and long dormancy process, here, we utilized “-omics” approach to obtain transcriptomes of two different germination stages of peony seeds. We extracted total RNA, sequenced the RNA using the Illumina/HiSeq™2000/MiSeq™ platform, assembled de novo, and annotated unigenes [14]. We identified *P. lactiflora* genes that were differentially expressed by seed hypocotyls during germination and analyzed their functions and the mechanism of differential expression. We ultimately aim to identify the key seed germination and dormancy genes to improve the breeding process.

## 2. Results and Discussion

### 2.1. Sequence Read Mapping

Based on RNA-seq data from stratification 0 day and stratification 40 days of *P. lactiflora* seeds before germination of down-hypocotyl (PDB) and after germination of up-hypocotyl (PDA), we obtained a total of 120,181,964 unigene sequences. 48% (58,107,876) and 5.2% (6,274,088) unigene sequences were present in RNAs of PDB and PDA, respectively. A total of 123,577 contigs were obtained from sequence assembly, and 30% of the contigs have the length larger than 2000 bases.

### 2.2. Gene Ontology (GO) Classification

**Figure 1:**
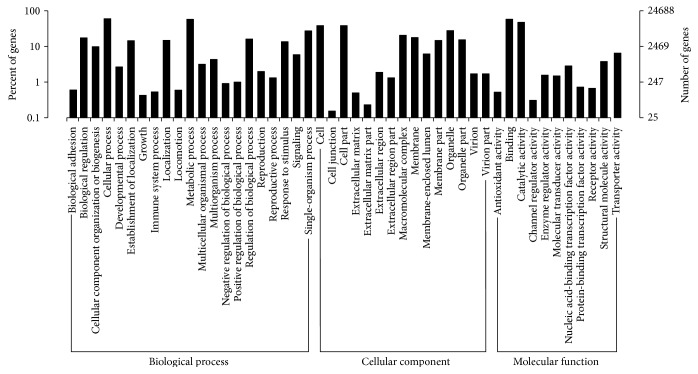
GO classification. *x*-axis represents the next level GO term of the GO three major categories. *y*-axis represents the number of genes annotated to the term (including the subterm) and the proportion to the total number of annotated genes. Three different GO categories include biological processes, cell components, and molecular functions.

The GO classification based on sequence homology revealed that 24,688 of the assembled unigenes were categorized into 47 functional groups (Figure 1). “Cellular processes” is the most abundant GO annotations (14,823, 21.78%), which included auxin response factors (*ARF*), ABA-responsive element-binding factors (*ABF*), brassinosteroid insensitive 1 (*BRI1*), and transcription factor TGA (*TGA*). “Binding” (14,364, 21.11%) was the second most prevalent category and included *ARF* and *BRI1*, followed by “metabolic processes” (14,261, 20.96%), including *ARF*, pyrabactin resistance/pyrabactin resistance-like (*PYR/PYL*), protein phosphatase 2C (*PP2C*), ethylene-insensitive protein 2 (*EIN2*), *ABF*, and *TGA*. Our annotation results show only a small proportion of the *P. lactiflora* unigenes with GO categories assigned, possibly due to the large number of uninformative gene descriptions of the plant protein hits. These classification results show the overall gene expression profile of *P. lactiflora* seeds.

### 2.3. EuKaryotic Orthologous Group (KOG) Classification

**Figure 2:**
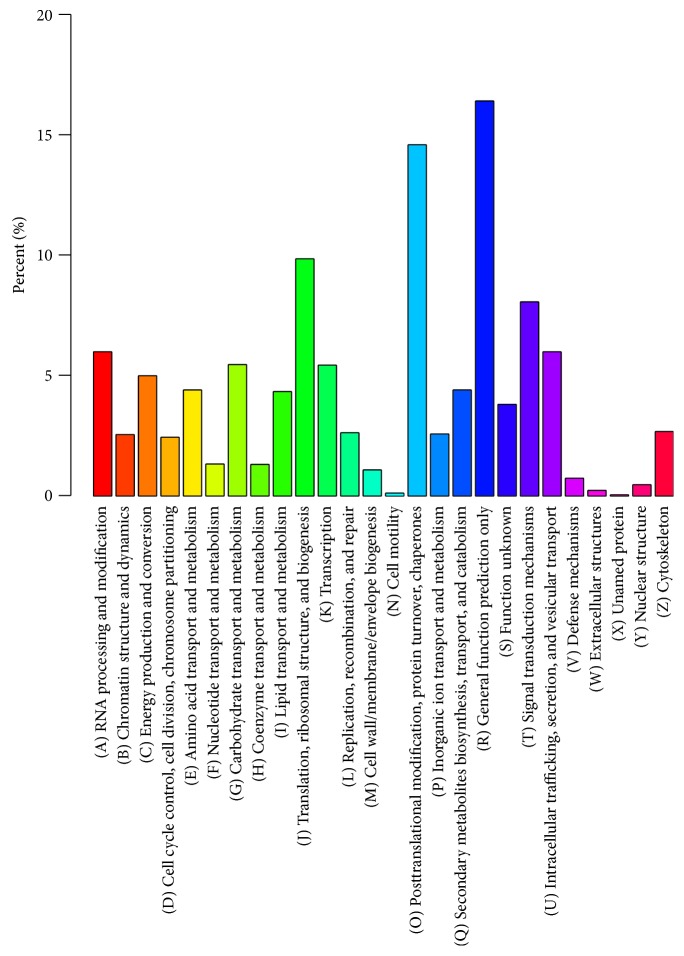
KOG classification. *x*-axis represents the name of the 26 groups of KOG. *y*-axis represents the ratio of the number of annotated genes for each group to the total number of annotated genes.

The KOG classification of 31,215 nonredundant hits indicated that 11,855 unigenes were clustered into 26 functional categories (Figure 2). “General functional prediction only” comprised the most common of the KOG annotations (1942, 16.38%), including coronatine-insensitive protein 1 (*COI1*), glycine-rich RNA-binding proteins (*GRP2*), and other genes, followed by “posttranslational modification, protein turnover, and chaperones” (1726, 14.56%), including S-phase kinase-associated protein 1(*SKP1*), inhibitors of invertases (*INH*), and other genes. The next most prevalent category was “translation, ribosomal structure, and biogenesis” (1164, 9.82%), including ribosomal protein L27b (*RPL27b*), ribosomal protein L13aa (*RPL13aa*), and other genes. The three rare groups were “unnamed proteins” (1, 0.0084%), “cell motility” (4, 0.034%), and “extracellular structures” (23, 0.20%).

### 2.4. Kyoto Encyclopedia of Genes and Genome (KEGG) Classification

**Figure 3:**
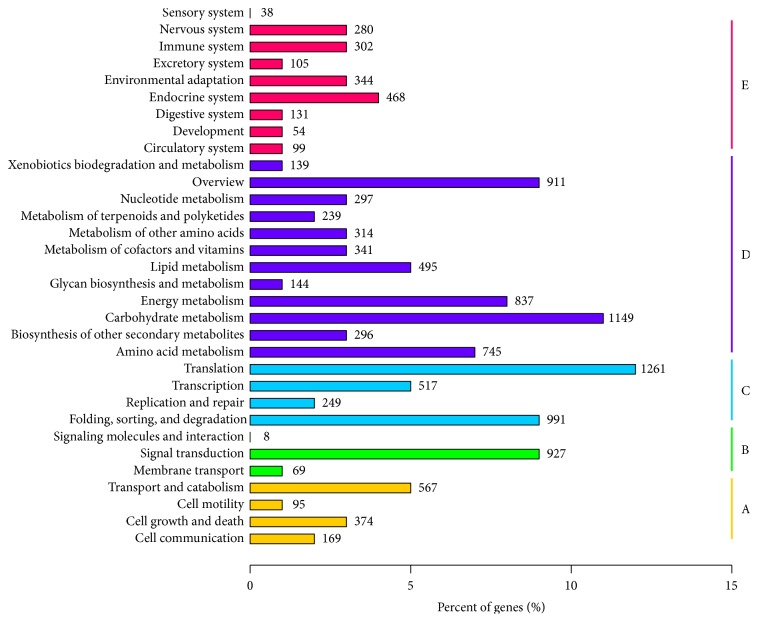
KEGG classification. *y*-axis represents the name of the KEGG metabolic pathway. *x*-axis represents the ratio of the number of genes to the annotated genes. The total number of genes for each pathway is shown on the top of each bar graph.

The KEGG analysis of unigenes showed that 10,714 unigenes were assigned to 258 pathways (Figure 3). The unigenes were divided into five branches according to the participating KEGG metabolic pathway: cellular processes (A), environmental information processing (B), genetic information processing (C), metabolism (D), and organismal systems (E). The major pathways containing hundreds of unigenes were “translation” (1261, 11.77%) followed by “carbohydrate metabolism” (1149, 10.72%) and “folding, sorting, and degradation” (991, 9.25%).

### 2.5. Simple Sequence Repeat (SSR) Analysis

A total of 9225 SSR loci were identified in the 68,054 contigs from *P. lactiflora* transcriptomes, accounting for 13.56% of all contigs. SSR types were abundant, including mononucleotide repeat to hexanucleotide repeat (Table 1). Among 6 types of SSR, mononucleotide repeat motifs are the most common, which account for 58.71%, following by dinucleotide repeat, trinucleotide repeat, tetranucleotide repeat, hexanucleotide repeat, and pentanucleotide repeat. These SSR markers are useful resources that can be utilized for the development of universal molecular markers and the construction of genetic map of *P. lactiflora.*

### 2.6. Prediction of Unigene Coding DNA Sequence (CDS)

**Figure 4:**
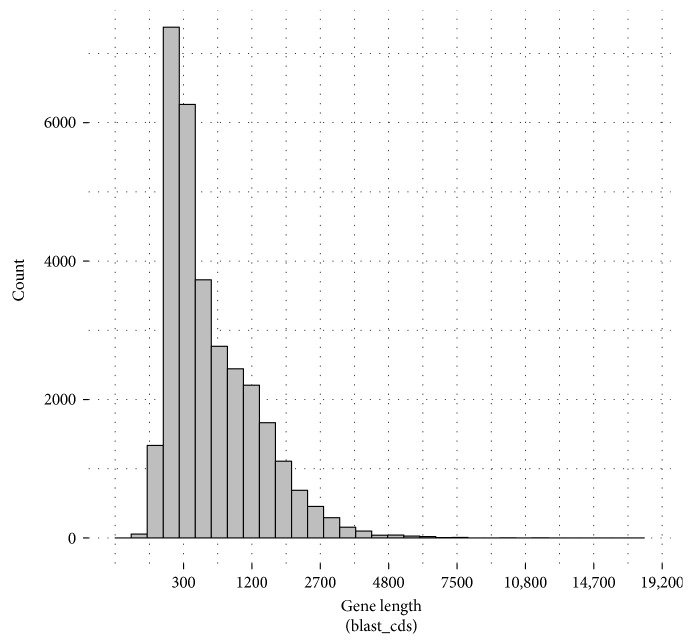
Length distribution of the predicted CDS.

**Figure 5:**
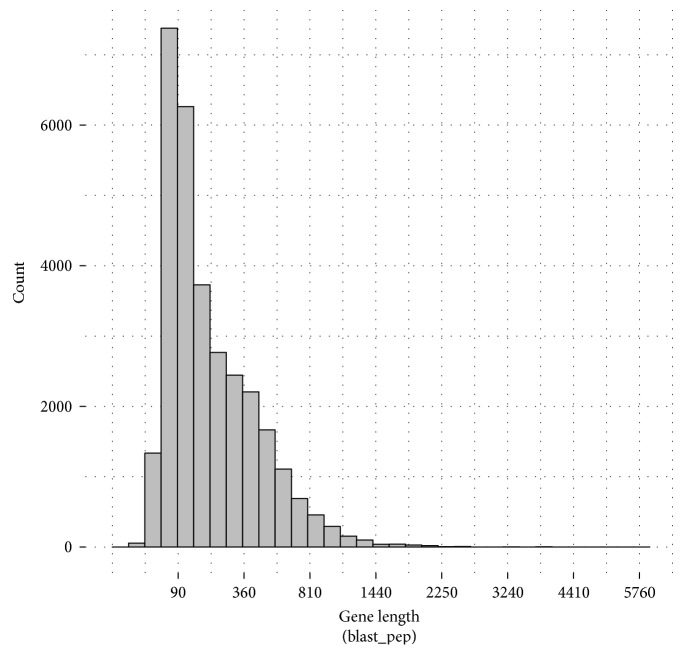
Length distribution of the predicted amino acids.

Compared with the NCBI nonredundant protein sequences (Nr), Swiss-Prot, KEGG, and KOG databases, we obtained that 30,803 unigenes contain CDS and encoded proteins. The lengths of predicted CDS are shown in Figure 4. The lengths of amino acids translated from predicted CDS are shown in Figure 5. Overall, we identified 8203 gene-predicted proteins with more than 300 (26.6%) amino acids and 745 gene-predicted proteins with more than 1000 (2.42%) amino acids.

### 2.7. Screening Differentially Expressed Genes (DEGs)

**Figure 6:**
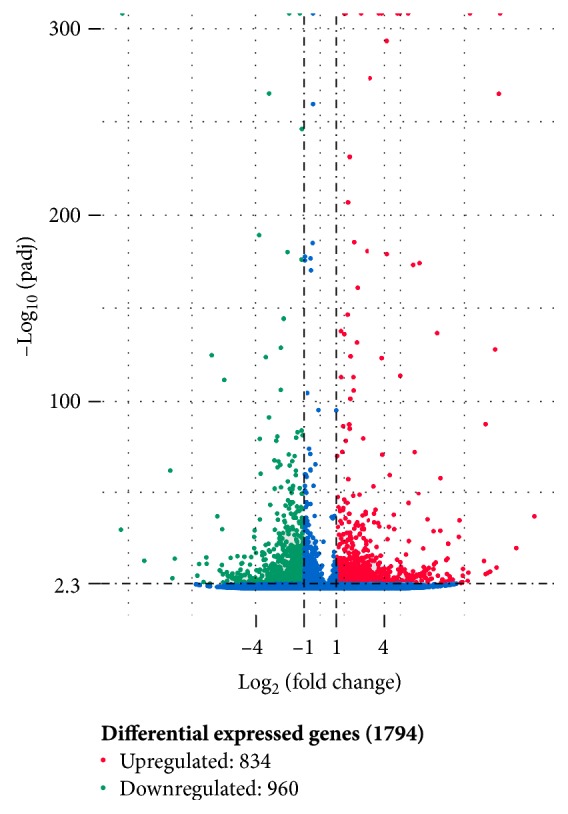
Analysis of DEGs between the experimental group and the sample (PDA versus PDB). Blue dots represent no difference between the genes of the two samples, red dots represent upregulated genes with significant differences, and green dots represent downregulated genes with significant differences.

The results of comparison of the two samples were quantitatively analyzed. According to the magnitude of gene expression, namely, expected number of fragments per kilobase of transcript sequence per million base pairs sequenced (FPKM), we calculated the multiple of gene expression difference between different samples. DEGs were defined with a threshold *q* value < 0.005 and │log_2_ (fold change)│ > 1. The statistical analysis results of the DEGs in the two samples are shown in Figure 6. The PDB sample had 834 upregulated and 960 downregulated genes relative to those in the PDA sample.

### 2.8. DEGs Significantly Enriched in the GO Functional Results

**Figure 7:**
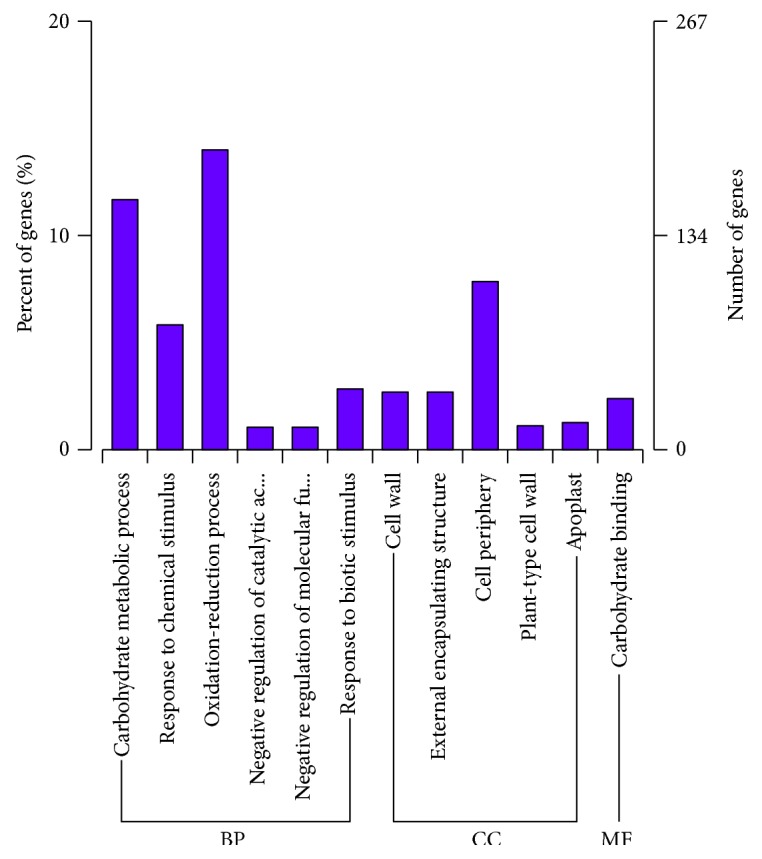
Enriched GO terms (PDA versus PDB). *x*-axis represents the next level GO term of the three major GO categories. *y*-axis represents the number of annotated genes for each term (including the subterm) and the proportion to the total number of annotated genes. Three different GO categories are biological processes, cell components, and molecular functions.

**Figure 8:**
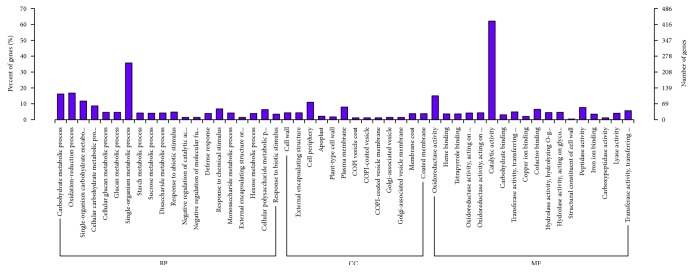
Upregulated genes of enriched GO terms.

**Figure 9:**
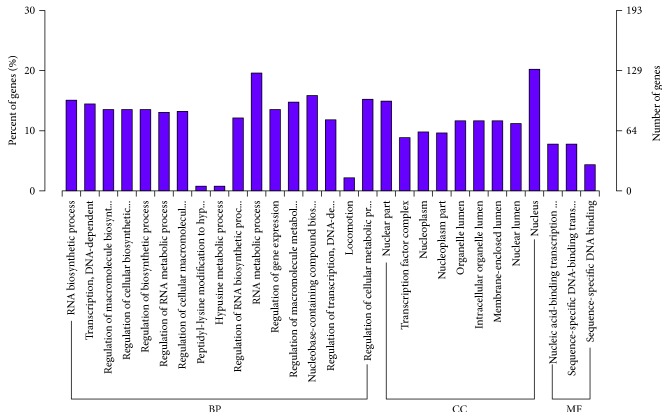
Downregulated genes of enriched GO terms.

We identified DEGs that were significantly enriched in GO entries and identified their biological processes, cellular components, and molecular functions at three levels of gene function (Figure 7). We enriched the analysis by using all DEGs for each combination and separately analyzed each combination of differences in the gene enrichment analysis based on up- or downregulation to better understand the gene functions (Figures 8 and 9).

“Oxidation-reduction processes” was the most significantly enriched biological process GO term for DEGs, accounting for 13.99%. “Cell periphery” was the most significantly enriched GO term in cellular components, accounting for 7.85%. “Carbohydrate binding” was the most significantly enriched GO term in molecular functions, accounting for 2.39%.

### 2.9. Significantly Enriched Pathways in the DEGs

In organisms, different genes are coordinated to exercise their biological functions. The most important biochemical metabolic pathways and signal transduction pathways of differentially expressed genes can be identified by the DEGs. The KEGG is the largest public pathway database [15]. We performed an enrichment analysis using the KEGG pathway unit and the hypergeometric test to identify the pathways for all annotated genes.

As the results of the analysis shown in Table 2, we observed 19 differential genes in the plant hormone signal transduction pathway (ko04075). Previous investigations reported that plant hormone signal transduction pathway (ko04075) is the plant hormone-regulating pathway, involving gibberellin, abscisic acid, cytokinin, auxin, ethylene and jasmonic acid, and other hormonal regulatory pathway [16]. Plant germination and growth is closely related to the regulation of hormones, so we lock this pathway as the research object.

### 2.10. Real-Time Polymerase Chain Reaction (PCR) Analysis of the Genes Involved in Plant Hormone Signal Transduction Pathways

**Figure 10:**
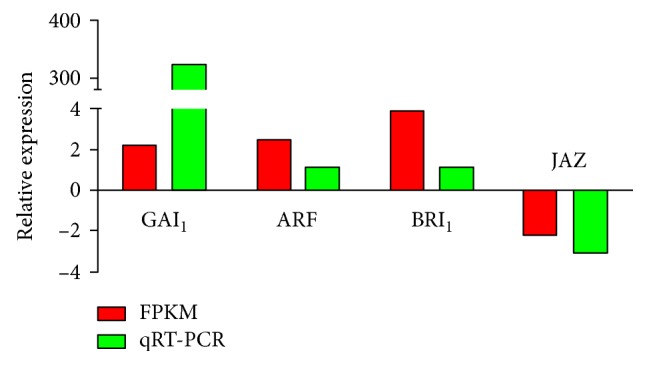
Quantitative real-time polymerase chain reaction validation of the fragments per kilobase of transcript per million mapped reads analysis of the four unigenes involved in plant hormone signal transduction in *Paeonia lactiflora* seeds.

Four plant hormone signal transduction unigenes were chosen for qRT-PCR analysis to confirm differences in expression levels between accessions found in the FPKM analysis. These unigenes were *GAI1*, *ARF*, and *BRI1*, which were upregulated in seeds, and *JAZ*, which was downregulated (Figure 10). The qRT-PCR data confirmed the expression patterns of these unigenes determined by the FPKM analysis.

## 3. Conclusion

This is the first study to apply RNA-seq transcriptomics profiling to investigate the sequences and transcript abundances of genes expressed in *P. lactiflora* seeds. This transcriptome analysis provided 68,054 unigenes, among which 45.86% were aligned to the Nr database, although no *P. lactiflora* reference genome sequence is available. The PDA and PDB identified a 1794 differentially expressed unigenes, including key dormancy and germination genes, such as *GAI_1_* and *ARFs*. *GAI_1_* inhibits elongation of Arabidopsis hypocotyl cells under dark and light conditions [17]. *ARFs* are transcription factors involved in auxin signaling pathway during many plant growth and developmental stages. *ARF10* mutant shows upregulation of ABA-responsive genes during germination [18]. The transcriptomes of *P. lactiflora* seeds provide us a basis for further exploration of *P. lactiflora* seed germination-related genes.

## 4. Materials and Methods

### 4.1. Materials

*P. lactiflora* was grown at Shenyang Agricultural University, Liaoning Province, China (41°80′N, 123°45′E) under field conditions. The male parent was “Fen Yulou,” and the female parent was “Fen Yunu.” We harvested the seeds annually every August and used two different seed developmental stages (0 and 40 days) as material. We extracted total RNA from seeds in the two stages and performed an RNA-seq transcriptome analysis.

### 4.2. RNA Extraction and Transcriptome Sequencing

Beijing Biological Information Technology Ltd. (Beijing, China) extracted total RNA, according to the manufacturer's instructions, which was stored at −80°C. Total RNA was extracted from seeds using the RNAprep pure Plant Kit (Tiangen Biotech, Beijing, China); each sample contains about ten seeds. We used the Illumina HiSeq™2000/MiSeq™ sequencer to sequence the *P. lactiflora* seed transcriptome [19]. The total extracted RNA was detected with the Agilent 2100 instrument (Agilent Technologies, Palo Alto, CA, USA) after passing sample testing using oligo (dT) magnetic bead-enriched eukaryotic mRNA. The constructed cDNA library was sequenced with the Illumina HiSeq 2000.

### 4.3. Raw Sequencing Data Processing

The image data file obtained by sequencing was converted into raw reads using CASAVA base calling. Joint reads were removed from raw reads, and an *N* ratio > 2% was used to remove the low-quality reads and obtain the clean reads. All subsequent analyses were based on the clean reads.

### 4.4. De Novo Assembly

Because *P. lactiflora* has no reference genome, we carried out de novo assembly using Trinity. First, the contigs were assembled using the overlapping area of the reads. Second, connected to the contig assembly sequence into the ends cannot be extended again (unigene). The unigenes were compared with the Nr, NCBI nonredundant nucleotide sequences (Nt), Swiss-Prot, KEGG, and KOG databases to determine the direction of the unigenes.

### 4.5. Unigene Functional Annotation, GO Classification, and Pathway Enrichment Analysis

The GO functional classification of the unigenes was performed using Blast2GO [20], and the pathway analyses were performed using the KEGG annotation service [21].

### 4.6. Screening of Differentially Expressed Unigenes, GO Classification, and Pathway Analysis

Prior to differential gene expression analysis, for each sequenced library, the read counts were adjusted by edgeR program package through one scaling normalized factor. Differential expression analysis of two samples was performed using the DEGseq (2010) R package. *P* value was adjusted using *q* value. *q* value < 0.005 and │log_2_ (fold change)│ > 1 were set as the threshold for significantly differential expression.

The analytic formula is

(1) $$\begin{array}{c} p=1-\overset{m-1}{\underset{i=0}{\sum }}\frac{\left(\begin{array}{c} M\\ i \end{array}\right)\left(\begin{array}{c} N-M\\ n-i \end{array}\right)}{\left(\begin{array}{c} N\\ n \end{array}\right)}. \end{array}$$

In the formula, *N* is the number of genes with pathway annotations in all genes; *n* is the number of differentially expressed genes in *N*; *M* is the number of genes annotated for a particular pathway in all genes; *m* is the number of differentially expressed genes for a particular pathway.

GO enrichment analysis of the DEGs was implemented by the GOseq R package-based Wallenius noncentral hypergeometric distribution [22], which can adjust for gene length bias in DEGs.

KEGG [23] is a database resource for understanding high-level functions and utilities of the biological system, such as the cell, the organism, and the ecosystem, from molecular level information, especially large-scale molecular datasets generated by genome sequencing and other high-throughput experimental technologies (http://www.genome.jp/kegg/). We used KOBAS [24] software to test the statistical enrichment of differential expression genes in KEGG pathways.

### 4.7. SSR Locus Search and Analysis

SSRs of the transcriptome were identified using MISA (http://pgrc.ipk-gatersleben.de/misa/misa.html), and primer for each SSR was designed using Primer3 (http://primer3.sourceforge.net/releases.php). The standard used for a SSR was 10 single-nucleotide repeats, six dinucleotide repeats, and three, four, five, and six nucleotides repeated at least five times [25].

### 4.8. Analysis of Unigene-Encoded Proteins (CDS)

The highest score from the BLAST (Basic Local Alignment Search Tool) alignment results was used to determine the CDS of the unigene, using the Nr, Swiss-Prot, KEGG, and Genes databases, in that order, to compare the unigenes. The transcripts were extracted from the comparative result of open-reading frame- (ORF-) encoding box information, and the standard table-coding region sequences were translated into amino acid sequences (5′-3′ order). The results obtained by comparing the known protein database with blast are shown in Supplementary File 1 available online at https://doi.org/10.1155/2017/8027626. If not, the ORF of the unigene was predicted using Estscan (3.0.3) software to obtain the nucleotide and amino acid sequences of this portion of the gene. The CDS results predicted by Estscan software are shown in Supplementary File 2.

### 4.9. Real-Time qRT-PCR Analysis

Four unigenes involved in plant hormone signal transduction were chosen for validation by qRT-PCR. The reference gene selected in this experiment was actin (Gene Bank query number is gi: 48927617). The primers were designed with Primer Premier 5.0 software (Table 3). Total RNA was extracted with the RNA prep pure Plant Kit and reverse transcribed into cDNA using the PrimeScritH RT reagent kit with the gDNA Eraser (Perfect Real Time) (Takara Bio, Dalian, China). qRT-PCR was performed with a Bio-Rad CFX-96 Real-Time PCR System (Bio-Rad, Hercules, CA, USA) in a final volume of 20 *μ*l containing 2 *μ*l cDNA, 10 *μ*l 26SYBR premix Ex taq™ (Takara Bio, Shiga, Japan), 0.4 *μ*l each of 10 mM forward and reverse primers, and 7.2 *μ*l RNase-free water. The thermal cycling conditions were 95°C for 5 min, 45 cycles at 95°C for 5 s for denaturation, and 56°C for 25 s for annealing and extension.

## Supplementary Material

Supplementary file 1: The result of alignment. Supplementary file 2: The result of prediction.

## Figures and Tables

**Table 1:** Microsatellite repeat motifs and their frequency of occurrence in the *Paeonia lactiflora* seed transcriptome.

Repeat type	SSR number	Proportion (%)	Repeat class
Mononucleotide repeat	5416	58.71	A/T
Dinucleotide repeat	2428	26.32	AG/CT
Trinucleotide repeat	1308	14.18	GGT/CCA
Tetranucleotide repeat	46	0.50	AAAC/TTTG
Pentanucleotide repeat	11	0.12	TATAT/ATATA
Hexnucleotide repeat	16	0.17	CTGTGG/GACACC

**Table 2:** Genes and their functions concentrated in the Kyoto Encyclopedia of Genes and Genomes (KEGG) pathways.

Term	Database	ID	Sample number	Background number	*P* value	Corrected *P* value
RNA transport	KEGG pathway	ko03013	30	292	0.000866	0.158438
Carbon metabolism	KEGG pathway	ko01200	43	488	0.001865	0.170690
Starch and sucrose metabolism	KEGG pathway	ko00500	25	254	0.003958	0.241449
Plant hormone signal transduction	KEGG pathway	ko04075	19	265	0.151777	1
Microbial metabolism in diverse environments	KEGG pathway	ko01120	49	654	0.019961	0.670138

**Table 3:** qRT-PCR validation primers.

Gene	Primer sequences(5′-3′)
Actin	GGTCTATTCTTGCTTCCCTC CCCTCTGCGTCTACACTTTC
GAI_1_	CAAGAAGCCAACCACAACGG TCACAAGCCACCACGTTACA
ARF	TGAGATTTGAGGGTGAGGAAG GGAGGAGGAGTTGTGGTATTG
BRI_1_	TGAAGCACTGAGCATCAACCTT TCACCAAAACCACCAGAACCAA
JAZ	AAACAAACCCTCCCCAACAG AACGCCACCAGGAACCATAG
