# Supplementary material for: Transcriptome Analysis of Two Different Developmental Stages of Paeonia lactiflora Seeds
Source: Int J Genomics. 2017 Aug 7;2017:8027626. doi: 10.1155/2017/8027626 (PMC5564112; doi:10.1155/2017/8027626)
Supplement: Supplementary file 1 — The result of alignment. Supplementary file 2: The result of prediction. [file 8027626.f1.pdf]

Supplementary File 1

The results obtained by comparing the known protein database with blast:

>comp32191_c0;orf1 len=1089 frame:-2 start:1302 end:214 PREDICTED: ell-associated factor Eaf isoform 1 [Vitis vinifera] >gi|147820042|emb|CAN62810.1| hypothetical protein VITISV_025292 [Vitis vinifera]

ATGGCGAACAATAATACGAGCAAAGAACCGAGCACTGCACCTGCGCCAGATCGGTGGTAT

AACCTAACCCTAGGTTCTTCCTTCAAAGACCACCAGCCATCCTCTAAATTCTGCACTTTA

CGATATGAATTTAAACCAGCTTCAATTGATAAGAGTCAACCCGGGTCATTAACCAAGAAC

AAGGAAAACAGGATCTCTGTAGAATTTCACAACAATCAACATGGAAAACCAAAAGTGACA

TTTGAGGGAAGCAGTGAGGACTACAAAGAAAATGACGCTGTTTTGTTTTTTGACGGTGAG

ACCTTTCGGTTGGAGAGGTTACACCGAGCAGTAAAGCGGTTGAGACATGTCCGACTTCCT

GGTGAATCTGCAGCTGCAAGTACCATGGCAGCGGGTACTTCTGTTGGACCAGCAGGAGAA

TCTTTTTCACCACCAGTTGGGAAATCAACAAAGCAGTTTCAGTATTCTAATAAAAGCACA

TTTCATCCAATGCCGGTTGAGGTTGAACGGATCGATGTTGTGGACTCAGAGAGCATTGGT

ACAAAACCTAAAAACGACAAGGTTATCGACCCTCCATCATCTCATCAAAAACCACCAACC

CCATTACCATCACCAGACCCTATGACACTCGAATTGGAGCAACATCTGGATATAATGAAC

GATGACGACGATGTTGATATTGATGGTTTCGAAACTGCAGAAAAAGGGAATAATAATACT

AATGCTGCTGCTGCTGCTGAAAAAGAAACCATCCAATCTGGTATTGATATCAACTTACCA

CAACAAAATGACACAGATGATGAGATAGCCGATGTTGATGTGAGCGATGACGATGAAGCA

GATAAAGGTCGTAATGCTGCTGAAGCGTTGAGAGCTCAGGTGAATGCTCAGGTGAATTCT

GAGCAGAGGTCACCGTCGAGGTCGAGGTCGAGCTCGAGTAGTAGTAGTAGTAGTGGGAGT

GATAGTAAGAGTAGCGGGAGCGGGAGCGGGAGTGGCAGTGGCAGTGGAAGTGGTAGCAGT

AGTAGCAGCGGCAGCGGCAGTGCCAGTGGTAGTGAAAGCAGTGACGATGACTCTGTCAAC

TCTATCTGA

Head notes: > (Sequence ID, it’s the unique identifier of this gene); (orf1, Prediction of ORF ID, some genes have two predicted ORF); len：(The length of the base of the ORF); frame：(The reading frame of the ORF in the original gene, "-" denotes negative chain); start：(The initial position of the ORF in the original gene); end：(The termination of the ORF on the original gene); (The description of the gene to the protein library)

Supplementary File 2

The CDS results predicted by Estscan software:

```
>comp26509_c0; len=81 start:1 end:80

XTGGTTGTCGGGGTAGGTGCAGTAGCACGAGTAATGTGCGAATACATTAGGCTGGACGAG

AGTTATGTTGCACTGTGTTGG

>comp31743_c0; len=354 start:427 end:74; minus strand

TCTCTAGTGTGGACTCGTATGCGTATCTCCGGTGAATAACTCGGCGTTGTCACGGGTTTG

GAAGAGAACAGGGGAGGAGGGTTTCGGTTCCCTCATACTCGCTACTCGAGACCAGAGGCC

GCCAAAATCAGCGAACCCAGGGATAACCGATCGGCGAATCGCGAGGCTCAAGCCAGACCG

GCAAACCATGGGGCTCTAGCTGAXCAGGCGAACCACAGGCCACCGATCAGCGAACCTACA

GGTTCAAGCCGATTGAGCGAACTCGCTAGCAAATTGGAGCCTGATGAAACTCTAGCAATA

TCAGTCATTAACGCCGACAAAGCAATTATTTTGCTTGATTTCAACAGAAATTAA
```

Head notes: > (Sequence ID, it’s the unique identifier of this gene); (a represents another ORF of the gene); len：(The length of the base of the ORF); start：(The initial position of the ORF in the original gene); end：(The termination of the ORF on the original gene); (minus strand represents the ORF in the original transcript is a negative chain)
